# Mapping malaria transmission foci in Northeast Thailand from 2011 to 2021: approaching elimination in a hypoendemic area

**DOI:** 10.1186/s12936-024-05026-6

**Published:** 2024-07-17

**Authors:** Kulchada Pongsoipetch, Rebecca Walshe, Suwanna Mukem, Tanong Kamsri, Navarat Singkham, Prayuth Sudathip, Suravadee Kitchakarn, Rapeephan Rattanawongnara Maude, Richard James Maude

**Affiliations:** 1 Mahidol Oxford Tropical Medicine Research Unit, Faculty of Tropical Medicine, Mahidol University, Bangkok, Thailand; 2 Faculty of Medicine, Ramathibodi Hospital, Mahidol University, Bangkok, Thailand; 3 Phibun Mangsahan Hospital, Ubon Ratchathani, Thailand; 4 Provincial Health Office, Ubon Ratchathani, Thailand; 5 Buntharik Hospital, Ubon Ratchathani, Thailand; 6 Division of Vector Borne Diseases, Department of Disease Control, Ministry of Public Health, Nonthaburi, 11000 Thailand; 7 Centre for Tropical Medicine and Global Health, Nuffield Department of Medicine, University of Oxford, Oxford, UK; 8 The Open University, Milton Keynes, UK

**Keywords:** Malaria, Surveillance, Thailand, Hot spot, Elimination

## Abstract

**Background:**

Thailand is approaching local elimination of malaria in the eastern provinces. It has successfully reduced the number of cases over the past decade, but there are persistent transmission hot spots in and around forests. This study aimed to use data from the malaria surveillance system to describe the spatiotemporal trends of malaria in Northeast Thailand and fine-scale patterns in locally transmitted cases between 2011 and 2021.

**Methods:**

Case data was stratified based on likely location of infection and parasite species. Annual Parasite Index per 1000 population (API) was calculated for different categories. Time series decomposition was performed to identify trends and seasonal patterns. Statistically significant clusters of high (hot spots) and low (cold spots) API were identified using the Getis-Ord Gi* statistic. The stability of those hot spots and the absolute change in the proportion of API density from baseline were compared by case type.

**Results:**

The total number of confirmed cases experienced a non-linear decline by 96.6%, from 1061 in 2011 to 36 in 2021. There has been a decline in both *Plasmodium vivax* and *Plasmodium falciparum* case numbers, with only four confirmed *P. falciparum* cases over the last two years—a 98.89% drop from 180 in 2011. API was generally higher in Si Sa Ket province, which had peaks every 2–3 years. There was a large outbreak in Ubon Ratchathani in 2014–2016 which had a high proportion of *P. falciparum* reported. The proportion of cases classified increased over the study period, and the proportion of cases classed as indigenous to the village of residence increased from 0.2% to 33.3%. There were stable hot spots of indigenous and imported cases in the south of Si Sa Ket and southeast of Ubon Ratchathani. *Plasmodium vivax* hot spots were observed into recent years, while those of *P. falciparum* decreased to zero in Ubon in 2020 and emerged in the eastern part in 2021, the same year that *P. falciparum* hot spots in Si Sa Ket reached zero.

**Conclusions:**

There has been a large, non-linear decline in the number of malaria cases reported and an increasing proportion of cases are classed as indigenous to the patient’s village of residence. Stable hot spots of ongoing transmission in the forested border areas were identified, with transmission likely persisting because of remote location and high-risk forest-going behaviours. Future efforts should include cross-border collaboration and continued targeting of high-risk behaviours to reduce the risk of imported cases seeding local transmission.

**Supplementary Information:**

The online version contains supplementary material available at 10.1186/s12936-024-05026-6.

## Background

The World Health Organization (WHO) set global targets to reduce malaria incidence and mortality rates by at least 90% by 2030, eliminate malaria in at least 35 countries by the same year, and prevent its resurgence in all malaria-free countries [1]. The elimination of malaria is defined as “the reduction to zero of local, or indigenous, malaria incidence” [2, 3]. The Greater Mekong Subregion (GMS), comprising Cambodia, Myanmar, Thailand, the Lao People’s Democratic Republic (Lao PDR), parts of the People’s Republic of China (Yunnan province and Guangxi Zhuang Autonomous Region), and Viet Nam, has the target of eliminating *Plasmodium falciparum* by 2025 and all forms of malaria by 2030 [4].

Thailand, with its previous goal to eliminate indigenous malaria by 2024, has been making significant progress but still faces ongoing challenges. The Division of Vector-borne Diseases (DVBD), Department of Disease Control, Ministry of Public Health, Thailand set an intermediate target of zero malaria transmission in 95% of districts by 2021. As of the 2019 fiscal year, 764 of 928 (88.7%) districts reported no local transmission [2]. The remaining pockets of endemicity in the other 164 districts were generally found in remote, forested border regions of Tak province bordering Myanmar to the west; Yala bordering Malaysia to the south; and Ubon Ratchathani and Si Sa Ket provinces in the northeast bordering Lao PDR and Cambodia [4]. Conflict in Myanmar on the western border has hampered local malaria control efforts [5] and there has subsequently been a large increase in cases in western Thailand since late 2021[6]. Emerging artemisinin and artemisinin-based combinations drug resistance [7] in the northeast has led to treatment failures, but numbers of cases in this area are now very low. Elimination of malaria from a region requires both draining the endemic parasite reservoir and minimizing local transmission around imported infections [8]. This necessitates effective surveillance which allows the identification of indigenous (locally transmitted) and imported cases.

The electronic Malaria Information System (eMIS) “Malaria Online” has been implemented nationwide since 2012 [9]. Between 2012 and 2015, DVBD’s malaria control efforts led to a significant decrease in the blood slide positivity rate (SPR) to < 5% among suspected fever cases [10] and an annual parasite index (API) < 1 per 1000 population (ranging from 0.38 to 0.82) [11]. A subsequent programme review in 2015 prompted a strategic shift from malaria control to elimination [11]. In 2016, the DVBD launched the National Malaria Elimination Strategy 2017–2026 and the complementary Malaria Elimination Operational Plan 2017–2021 to be implemented at the local level. Inspired by the 1-3-7 strategy successfully used in China to reach malaria elimination status, the focus of these plans was to “identify infections rapidly and to use timely and active surveillance and response to prevent them from spreading” [2]. Under the 1-3-7 strategy, cases are reported within 1 day, investigated within 3 days, and foci are investigated and responded to within 7 days [10, 12]. The DVBD also upgraded the eMIS to consolidate all sources of malaria case data and allow monitoring of routine surveillance data and activities in near real-time [9]. Malaria cases in Thailand are classified by the likely location of infection based on case investigation and reported travel history [10], with the aim of differentiating local transmission from imported malaria cases. This classification is key to informing the DVBD’s decision-making on where to prioritize malaria interventions, such as case detection and treatment, vector control, surveys and research, and disease surveillance [2].

Here the aim was to describe the spatiotemporal trends of malaria incidence in two provinces in Northeast Thailand using high-quality surveillance data which covers a dynamic 10-year time period. The general trend in cases and the proportion classified by the surveillance system are described followed by the hot spots of local transmission and imported cases as well as *P. vivax* and *P. falciparum* infections at subdistrict and village levels, and mapping the stability of these hot spots and the change in malaria incidence relative to baseline. By mapping the stable foci of endemic transmission and the sites at which local transmission occurs, the aim was to help inform targeted, efficient use of resources as Thailand approaches elimination.

## Methods

### Study setting

**Fig. 1:**
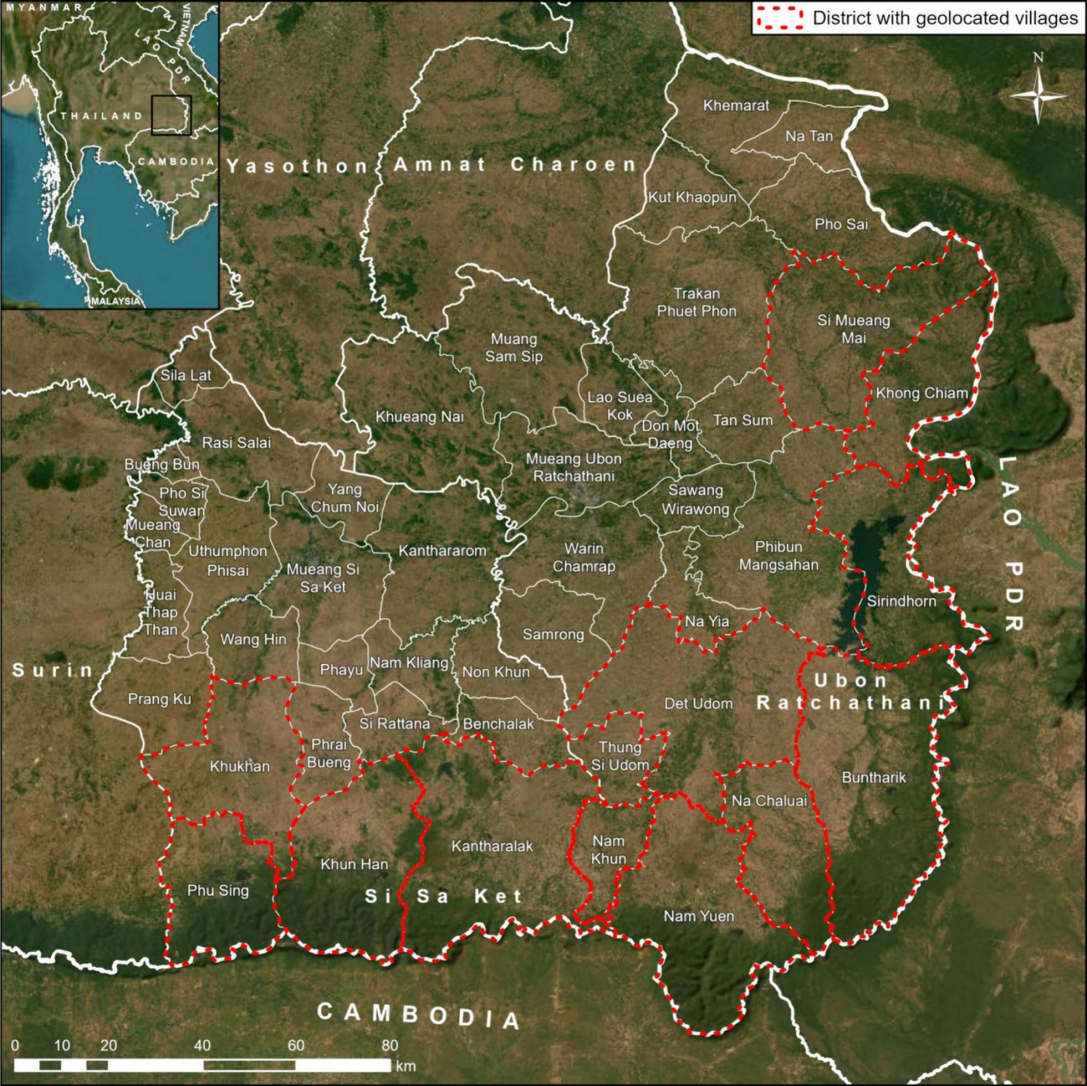
Map of the study area in Northeastern Thailand. Districts where all villages were geo-located are outlined in red

The study areas were Si Sa Ket and Ubon Ratchathani provinces in Northeast Thailand (Fig. 1). The provinces are divided into 22 and 31 districts, respectively. Both provinces share a forested southern border with Cambodia, and Ubon Ratchathani borders Lao PDR to the east.

### Village population and GPS coordinates

Population counts per village per year from 2011 to 2021 were accessed from data published online by the Administration and Registration Technology Development Division, Bureau of Registration Administration, Department of Provincial Administration, Ministry of Interior [13]. This data was combined to create a list of 5446 villages in Si Sa Ket and Ubon Ratchathani with their annual population counts which were aggregated at subdistrict level to calculate API, as detailed in Additional file 1. For village level analysis, GPS coordinates were added manually as they were not included in the village population data. For this, only villages in districts contributing 95% of all malaria cases in their province were included. The sources used for acquiring village GPS coordinates are described in Additional file 1. A total of 1640 villages were geolocated, covering 29.79% (786 villages) of all villages in Si Sa Ket and 31.58% (854 villages) in Ubon Ratchathani, representing all villages in four of 22 and eight of 31 districts in those provinces, respectively (Fig. 1).

The dataset of Thailand’s administrative boundaries from the Royal Thai Survey Department [14] was used for mapping other administrative levels of Thailand. The country boundaries published by the Database of Global Administrative Areas version 4.1 [15] were used for mapping other countries (Fig. 1).

### Malaria surveillance data

Individual anonymized records of malaria cases reported between 2011 and 2021 in Si Sa Ket and Ubon Ratchathani were obtained from the DVBD. In Thailand, most malaria cases are diagnosed through passive case detection by rapid diagnostic test (RDT) and light microscopy at malaria posts, health-promotion hospitals, and district hospitals. A confirmed case was any person with a positive malaria blood smear or RDT result reported by government health workers [2]. The data also included actively detected cases from the investigation of transmission foci, which has been performed in Thailand since 2009 [10].

Cases were excluded from the subdistrict and village-level analyses where the village data was incomplete, as detailed in Additional file 1. The raw data contained 7942 cases in Si Sa Ket and 16,283 cases in Ubon Ratchathani from 2011 to 2021. Following the exclusion of cases with incomplete village location data, 7825 cases (98.53%) in Si Sa Ket and 15,745 cases (96.69%) in Ubon Ratchathani were included in the full analysis.

For the subdistrict-level analysis, all subdistricts in the two provinces were included and cases were aggregated by subdistrict and blood draw year to give the total number of cases per subdistrict per year. At this level, indigenous transmission was considered to be within the subdistrict of residence, while non-indigenous transmission occurred outside of the subdistrict. For the village-level analysis, the cases were aggregated by village and blood draw year. Indigenous transmission was considered to have occurred within the village of residence only.

Individual village/cluster risk stratification data in Si Sa Ket and Ubon Ratchathani between 2011 and 2021 fiscal years were provided by the DVBD. As well as classifying individual cases by site of infection, the DVBD also stratifies villages or clusters of houses in villages into risk groups based on recent indigenous malaria cases [2]. In this study, the presence of indigenous malaria transmission based on the risk stratification data is also discussed.

### Analysis

Spatial and temporal trends in malaria incidence were examined at the province, subdistrict, and village levels. The case data was analysed in three groups by likely location of infection: all classifications, indigenous cases only, and non-indigenous only; and in two groups by parasite species: *P. vivax* and *P. falciparum*. API was calculated as the number of confirmed cases per 1000 total population. The API for each year was calculated for each subdistrict and village.

### Temporal trend

Time series decomposition of monthly parasite incidence was performed at the province level for each case type using moving averages, with the decompose() function in R version 4.22 [16]. An additive seasonal component was assumed as this gave the smallest residuals.

### Hot spots

Hot and cold spots of API of each classification (all cases of malaria and indigenous cases only) and parasite species were identified each year at the subdistrict and village levels, and non-indigenous cases at the village level only, using the Getis-Ord Gi* statistic [17], as detailed in Additional file 1. At the subdistrict level, the analysis was performed using the local_gstar_perm() function in the sfdep package in R. The spatial weights among subdistricts were determined by geographical contiguity. Village-level analysis was performed in ArcGIS Pro version 3.1.0 [18] using the Hot Spot Analysis (Getis-Ord Gi*) tool. Inverse distance was specified as the spatial relationships among villages. The distance bandwidth was set to 8547 m, which was the maximum distance at which a village has at least one neighbour.

### Village hot spot stability

For each classification (all cases, indigenous and non-indigenous) and parasite species, the stability of hot spots was represented by the proportion of the density of years from 2011 to 2021 that a village was a hot spot with 95% or 99% confidence. Additional information on the analysis is provided in Additional file 1.

### Absolute change in the proportion of API density from baseline

Absolute change in the proportion of API density from baseline for each classification (all cases of malaria and indigenous cases only) and parasite species was represented by the proportions of API density relative to the baseline values. As the study used malaria data from 2011 onwards, the proportions of API density for each case type in 2011 were used as the baselines. Additional information on the analysis is provided in Additional file 1.

## Results

### Overview of cases

**Fig. 2:**
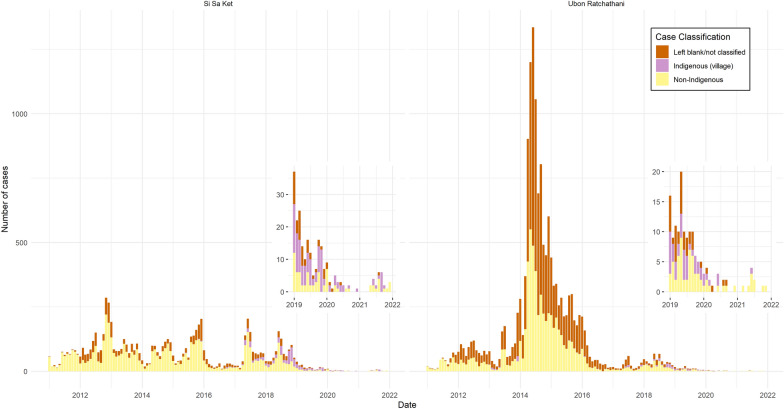
Number of monthly cases stratified by classification. The insets magnify case numbers from 2019 onwards. In this graph, indigenous refers to cases recorded as transmitted within the village, and non-indigenous anywhere outside of the village

**Fig. 3:**
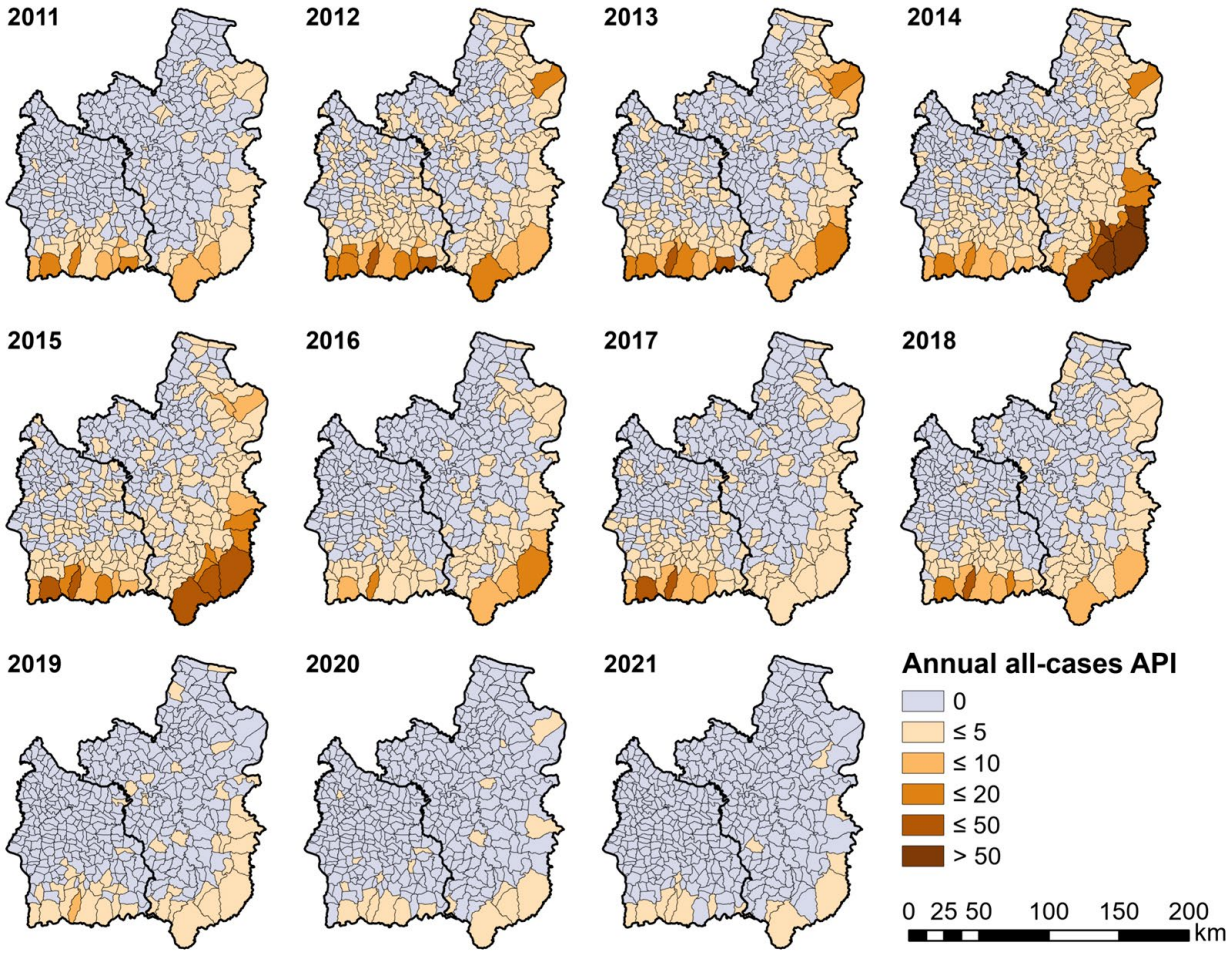
Annual all-cases API by subdistrict

The annual number of malaria cases across the two provinces declined by 96.6% from 2011 to 2021, from 1061 to 36 (see Additional file 2 for numbers by year, species and province). This decrease was non-linear (Fig. 2), with a peak of 9219 reported cases in 2014, the majority of which were in Ubon Ratchathani (Ubon). In 2014, there was a cross-border malaria outbreak between Lao PDR and Ubon [19], with a large peak of cases mainly concentrated in Buntharik, Na Chaluai, and Nam Yuen districts in the southeast of Ubon (Fig. 1). In some subdistricts of Buntharik and Na Chaluai, the all-cases API for 2014 exceeded 50, peaking at 159.17 in Huai Kha subdistrict of Buntharik (Fig. 3; southeasternmost subdistrict of Ubon). The overall annual number of cases in Si Sa Ket and Ubon dropped by 51.6% the following year. In 2017, an outbreak of predominantly *P. vivax* cases emerged in Si Sa Ket, primarily affecting Phu Sing, Khun Han, and Kantharalak districts along the border with Cambodia (20). The all-cases API for 2017 exceeded 20 in Dong Rak subdistrict of Phu Sing and Huai Chan subdistrict of Khun Han (Fig. 3; darker-coloured subdistricts, left to right). In recent years, malaria cases in the region have markedly declined, with most subdistricts reporting an API of zero, or fewer than five, since 2019. Only four cases of *P. falciparum* were reported between 2020 and 2011, a drop of 98.89% from 180 cases in 2011 (Additional file 2).

Both the number and proportion of cases classed as indigenous to the village of residence increased over time, while the number of cases not able to be classified or left blank reduced over time across both provinces (Fig. 2). In the past three years, Si Sa Ket had a higher proportion and number of indigenous cases than Ubon. Overall, cases classed as indigenous to the village of residence increased from two (0.2% of all cases) in 2011 to 12 (33.3%) in 2021.

**Fig. 4:**
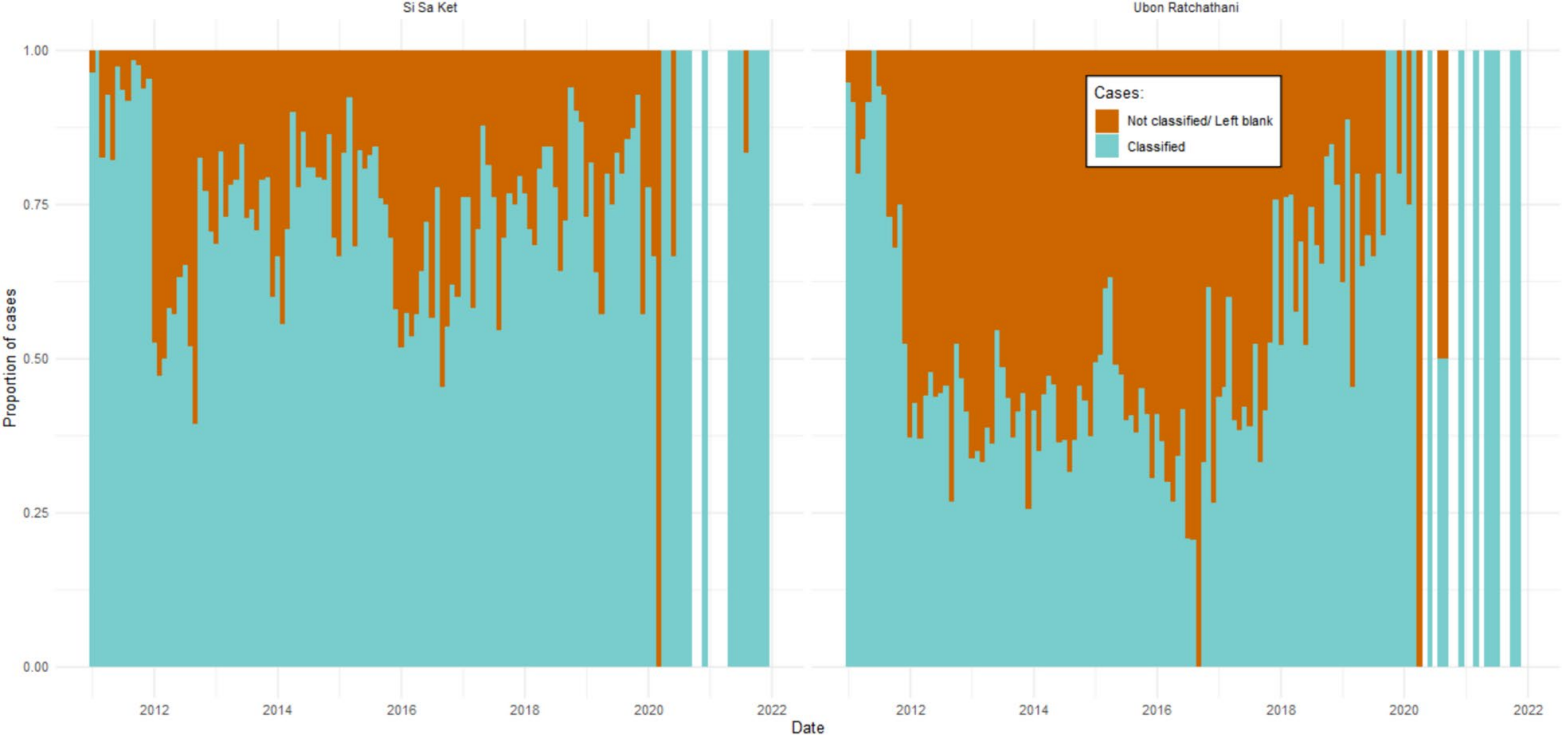
The proportion of reported cases over time which were labelled with a classification. The unfilled bars in 2021 represent months in which no cases were reported

**Fig. 5:**
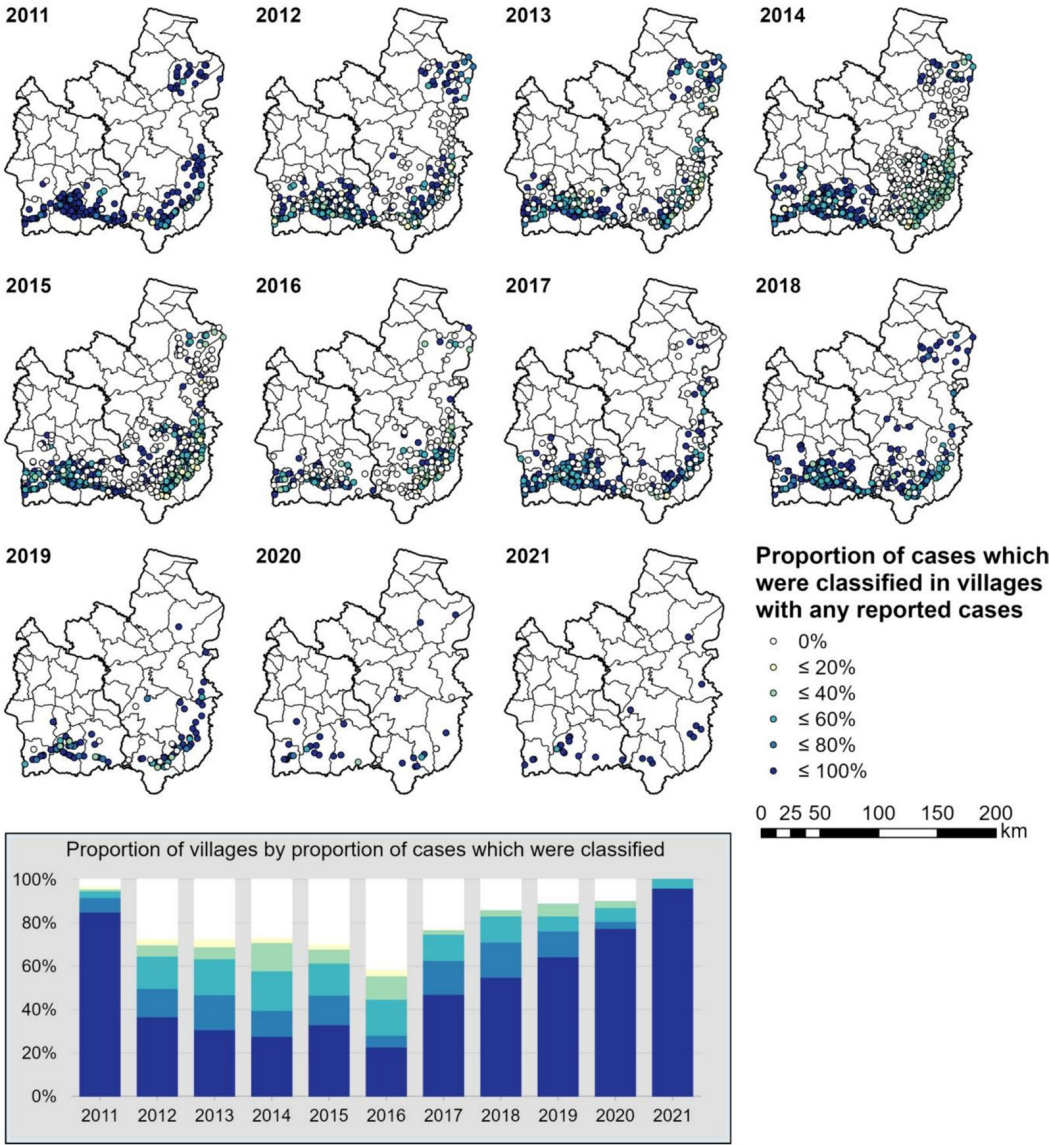
Proportion of cases which were classified annually in villages with any reported cases. The graph summarizes the proportion of mapped villages by the proportion of cases which were classified each year. The same classes and colour ramp as the map apply

The proportion of classified cases was initially high in 2011 (Fig. 4; > 75% in all months across both provinces) before a steep drop to around 50% in 2012. The classification proportions were particularly low in Ubon during and after the 2014 outbreak. The proportion classified has been subsequently increasing since 2017, and in 2021 97.2% (35 of 36) of reported cases were classified (Fig. 4). The proportion of classified cases per village is shown in Fig. 5. The number of villages with > 80% of cases classified has been increasing gradually since 2017, and in 2021 24 of 25 (96%) villages classified > 80% of cases. In 2014, however, 148 (26.8%) villages had 0% of cases classified, 87.8% of which were in Ubon. The proportion of villages with 0% of cases classified increased to 29.8% in 2015 and reached a peak of 41.5% in 2016. Of these villages, 74.5% and 66.1% were in Ubon, respectively.

### Seasonal trend

**Fig. 6:**
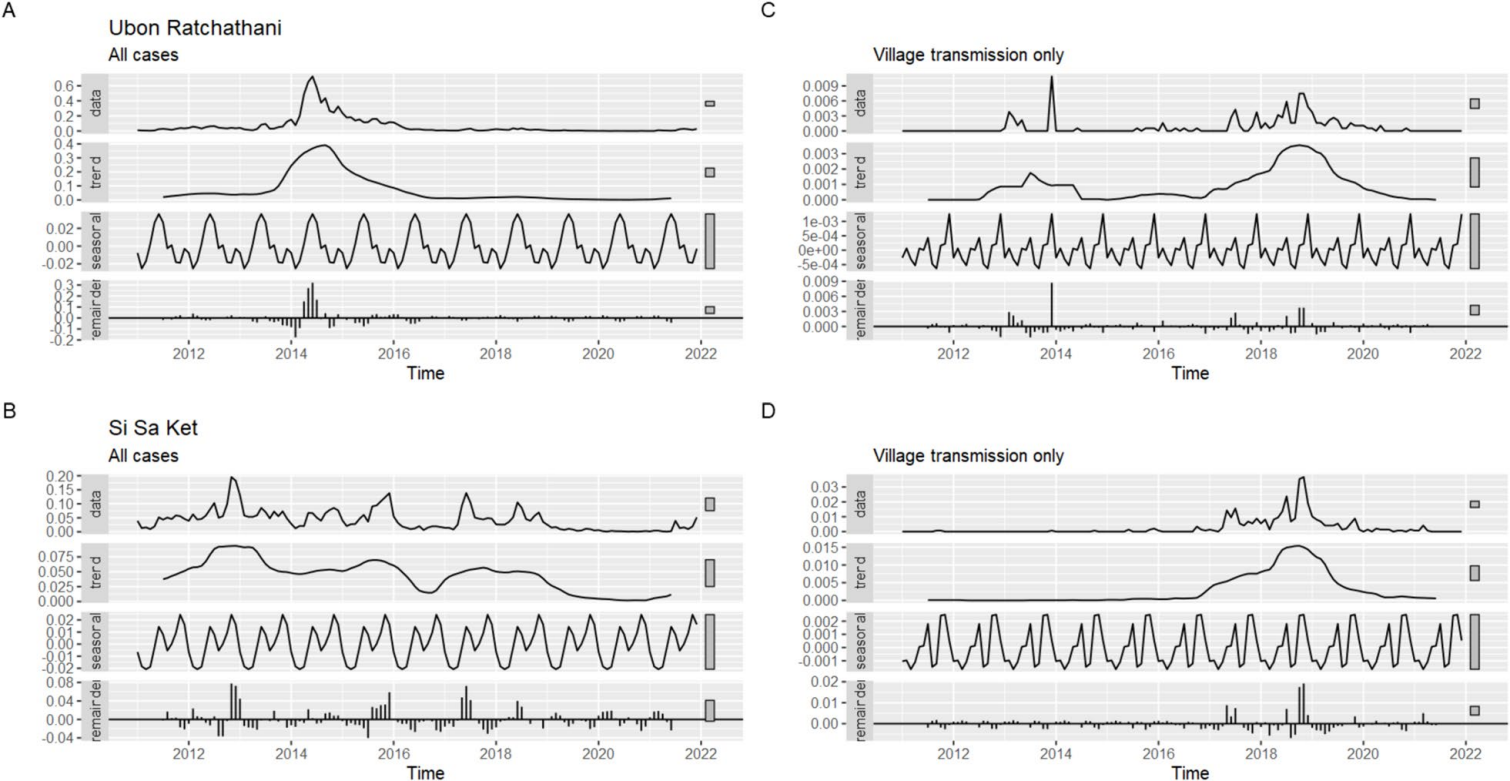
Province-wise time series decomposition of API for (**A** + **B**) all cases and (**C** + **D**) village transmission only. Upper panels are for Ubon Ratchathani while lower are for Si Sa Ket. An additive seasonal component is assumed. Note the different y axes

The all-case API was mostly higher in Si Sa Ket, except during the 2014 outbreak in Ubon (Fig. 6A, B). Ubon and Si Sa Ket have had different but overall negative trends in API. There were smaller peaks every 2–3 years in Si Sa Ket and a large peak in 2014–2015 in Ubon. The trend has been more negative overall in Si Sa Ket but with large fluctuations which were not explained by annual seasonality. Except for the 2014 outbreak, the trend in Ubon was relatively flat.

When including only indigenous cases classed as having been transmitted within the village of residence, the API was higher in Si Sa Ket than in Ubon (Fig. 6C, D). Both provinces had an extended peak between 2017 and 2020, but it was of lower magnitude and consistency in Ubon. Apart from this, the trend of indigenous cases was overall flat in both provinces.

Throughout the study period, the API for *P. vivax* was higher than *P. falciparum*. Both had similar seasonal trends and an overall decrease over time. In 2011, there were 881 *P. vivax* cases and 180 *P. falciparum*, declining to 33 and two cases, respectively in 2021. There was a large peak in 2014–2015 in which *P. falciparum* made up a much larger proportion of cases than during other years.

### Hot spots

**Fig. 7:**
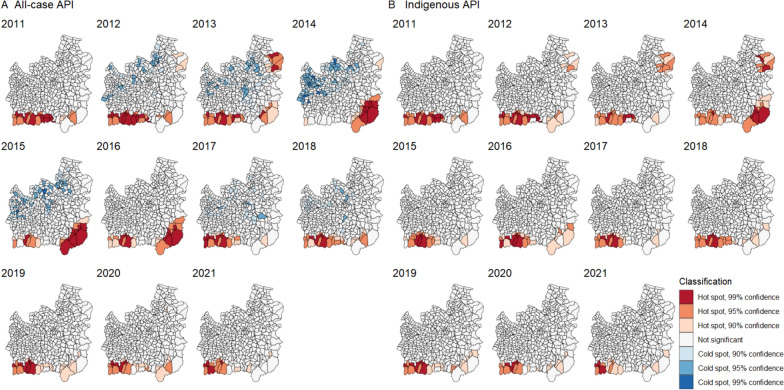
Subdistrict hot spots of (**A**) all-cases and (**B**) indigenous API. The hot spots were identified using the Getis-Ord Gi* statistic. Geographical contiguity was used as the spatial weights among subdistricts

There was a stable cluster of hot spot subdistricts along the southern border of Si Sa Ket for all malaria cases (Fig. 7A). This persisted throughout the whole study period but reduced in confidence during the outbreak in the southeast of Ubon, which is represented by high-confidence hot spots between 2014 and 2016. There were some cold spots in non-border subdistricts in both provinces, the confidence of which reduced over time.

Considering only cases indigenous to the subdistrict of residence, hot spots in the south of Si Sa Ket persisted throughout the study period (Fig. 7B). The hot spots in the southeast of Ubon in 2014 to 2016 were fewer and of lower confidence than for all cases, indicating that imported or unclassified cases contributed significantly to the all-cases hot spots there. This is consistent with the case mix shown in Fig. 2. There were also high-confidence hot spots in the northeast of Ubon in 2013 and 2014 which did not persist in later years.

**Fig. 8:**
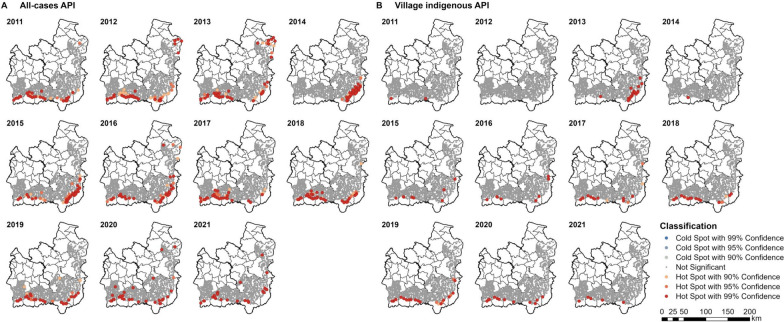
Village hot spots of (**A**) all-cases and (**B**) indigenous API. The hot spots were identified using the Getis-Ord Gi* statistic. Inverse distance was set as the spatial relationships among villages, with a threshold of 8547 m (the maximum distance of a village from at least one neighbour). Upon excluding cases with incomplete village data, no village indigenous cases in 2012 were included

No cold spots were identified when the hot spot analysis was performed at the village level (Fig. 8) in the 12 districts with geolocated villages (per Fig. 1). There was a similar geographic pattern to all cases at the village level (Fig. 8A) compared to the subdistrict (Fig. 7A), except that some villages in the northeast of Ubon were identified as hot spots in 2016, 2020, and 2021, when there was no subdistrict identified as a hot spot. Furthermore, although the subdistrict-level hot spots of API in Ubon decreased in number and confidence since 2017, some village-level hot spots remained highly significant.

There were very few village indigenous hot spots between 2011 and 2016, except for a cluster of villages in the southeast of Ubon in 2013 (Fig. 8B). There were low rates of case classification at the time: in 2014, 56.1% of cases were not classified, while cases classed as having been infected in the village made up only 0.02% (two cases; one in Khun Han district in Si Sa Ket and one in Tan Sum district in Ubon). From 2011 to 2021, only 2.9% of cases were classed as having been acquired within the village of residence, while 22.5% were acquired outside of the village but within the subdistrict of residence. Over time, the overall proportion of cases indigenous to the village of residence has increased up to a peak of 40.9% of all cases in 2019. This corresponded with an increase in the proportion of cases which were classified by likely location of infection (Fig. 4).

**Fig. 9:**
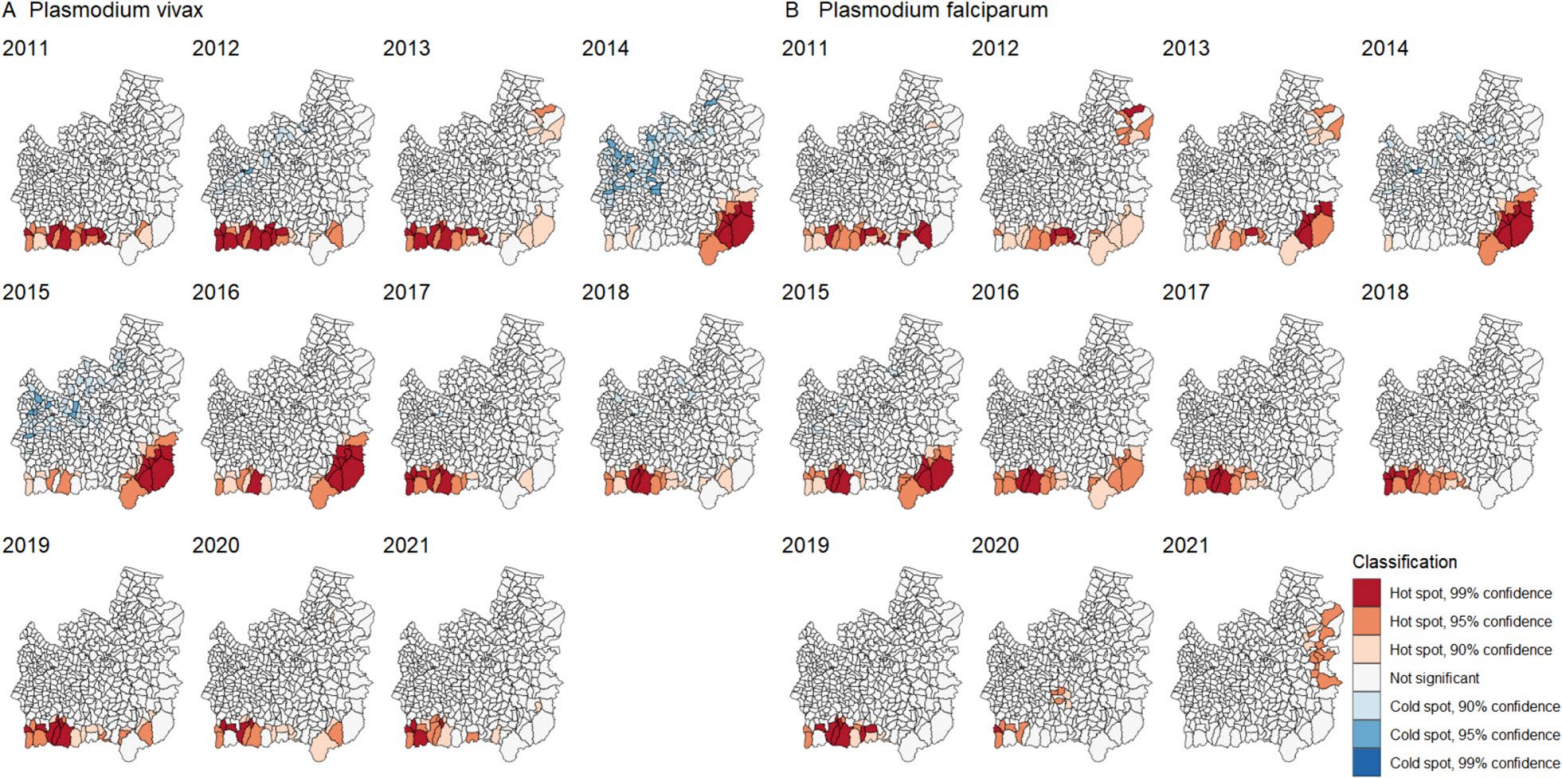
Subdistrict hot spots of *Plasmodium* (**A**) *vivax* and (**B**) *falciparum* API. The hot spots were identified using the Getis-Ord GI* statistic. Geographical contiguity was used as the spatial weights among subdistricts

**Fig. 10:**
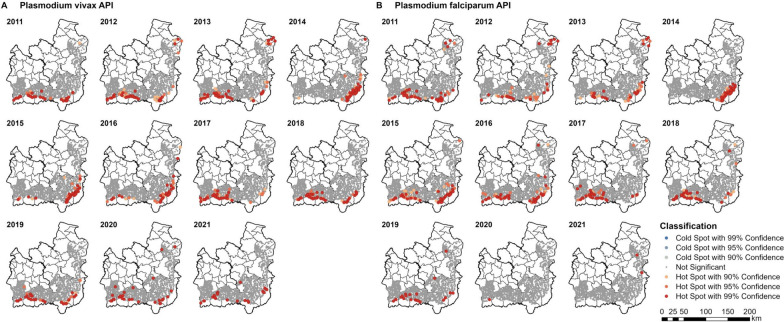
Village hot spots of *Plasmodium* (**A**) *vivax* and (**B**) *falciparum* API. The hot spots were identified using the Getis-Ord Gi* statistic. Inverse distance was set as the spatial relationships among villages, with a threshold of 8547 m (the maximum distance a village has to at least one neighbour)

Similar to the hot spot analyses by case classification, the subdistrict and village hot spots of *P. vivax* and *P. falciparum* API were generally close to the forested areas along the international borders of Si Sa Ket and Ubon (Figs. 9, 10).

The subdistrict hot spots of *P. vivax* API in the south of Si Sa Ket persisted into 2020 and 2021 (Fig. 9A). There have been no *P. vivax* hot spots on the northeastern border of Ubon since 2013. The *P. falciparum* hot spots in the south of Si Sa Ket persisted until 2020, when they became fewer (Fig. 9B). However, some *P. falciparum* hot spots on the eastern border of Ubon were found in 2021, where they had not previously been detected.

Although the number of village hot spots of *P. vivax* API reduced over time, several hot spots with 99% confidence still remained in place across much of the two provinces in 2020 and 2021 (Fig. 10A). There was a marked decline in the number of *P. falciparum* hot spots in Ubon since 2017, reaching zero and two hot spots in 2020 and 2021, respectively (Fig. 10B). The decline was seen in Si Sa Ket thereafter, to only one hot spot in 2020 and none in 2021.

### Village hot spot stability

**Fig. 11:**
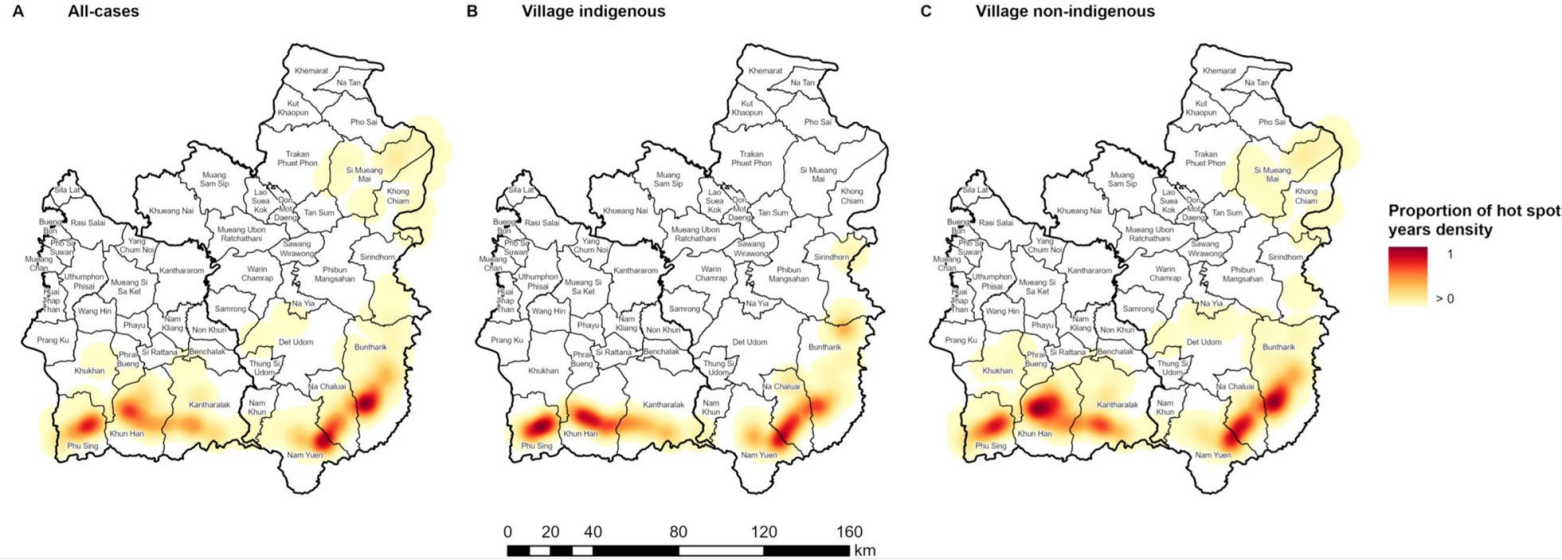
The stability of village hot spots of (**A**) all-cases, (**B**) indigenous, and (**C**) non-indigenous API. The stability was represented by the proportion of the density of years from 2011 to 2021 a village was a hot spot of API with 95% or 99% confidence. For each case classification, the proportion of hot spot years density was calculated by dividing each cell value by the maximum density value for that case classification

**Fig. 12:**
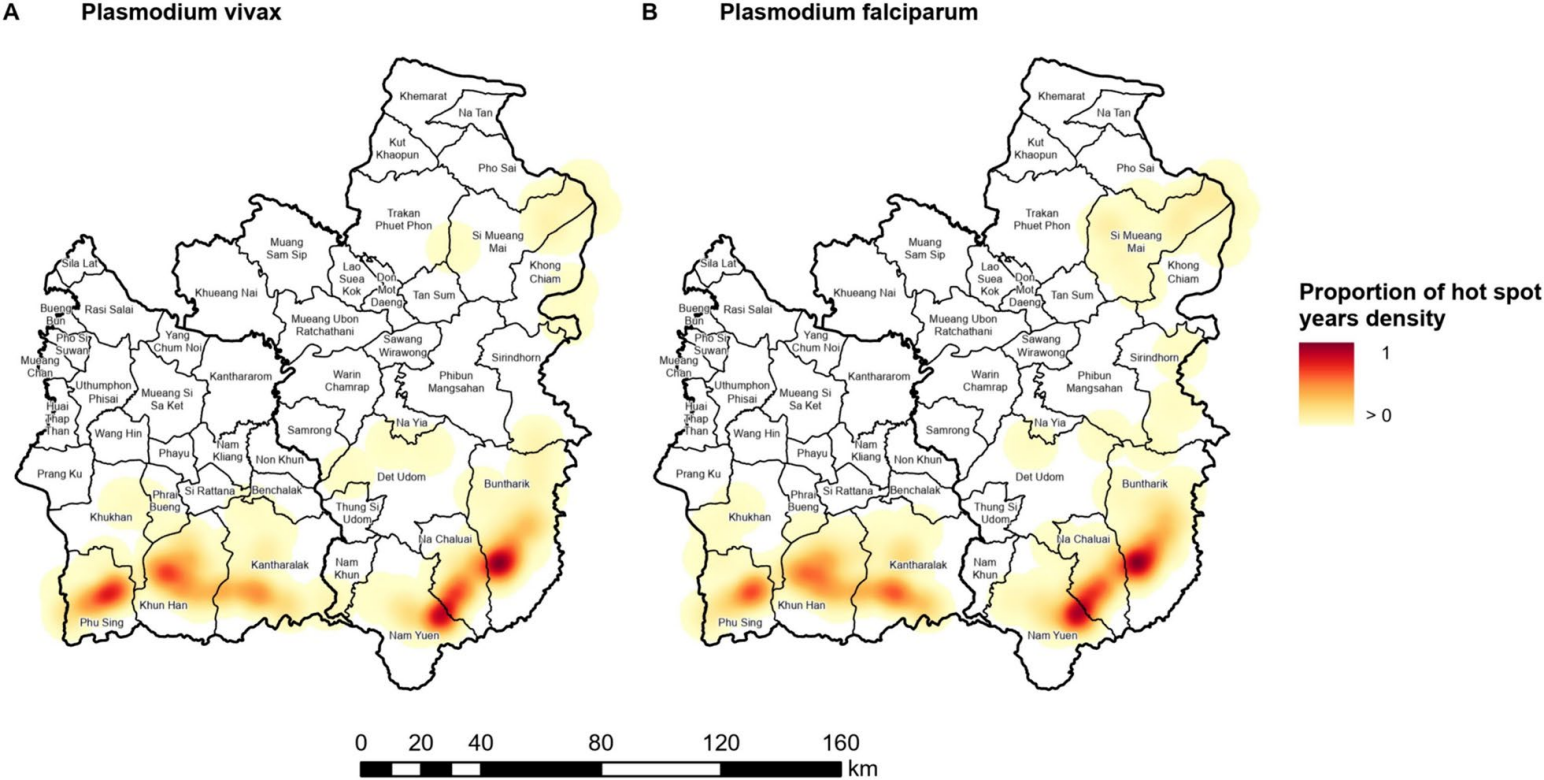
The stability of village hot spots of *Plasmodium* (**A**) *vivax* and (**B**) *falciparum* API. The stability was represented by the proportion of the density of years from 2011 to 2021 a village was a hot spot of API with 95% or 99% confidence. For each parasite species, the proportion of hot spot years density was calculated by dividing each cell value by the maximum density value for that parasite species

Regardless of case classification, village hot spots were located in the south of Si Sa Ket and southeast of Ubon throughout the study period (Fig. 11A)*.* The indigenous hot spots were particularly confined to areas close to the border and were most stable in Phu Sing and Khun Han districts in Si Sa Ket, and Nam Yuen and Na Chaluai in Ubon (Fig. 11B). Non-indigenous hot spots were also seen most frequently in these areas, but some less stable hot spots were also found in other districts further from the southern border (Fig. 11C). The distributions of hot spots for each of *P. vivax* and *P. falciparum* were very similar and also remained highly stable in the areas close to the forested borders (Fig. 12).

### Absolute change in the proportion of API density from baseline

**Fig. 13:**
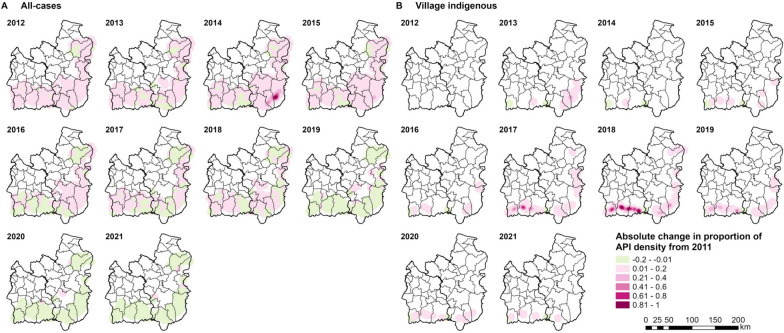
Absolute change in proportion of (**A**) all-cases and (**B**) village indigenous API density from 2011. For each case classification, the proportion of API density was calculated by dividing each cell value by the maximum density value for that case classification. Upon excluding cases with incomplete village data, no village indigenous cases in 2012 were included

Most parts of Si Sa Ket and Ubon saw an increase in the proportion of all-case API density from baseline between 2012 and 2015, with a maximum increase of 0.98 during the 2014 outbreak in Ubon and 0.29 in 2015, both in Buntharik district (Fig. 13A). In 2016, the proportion of API density dropped below baseline for many parts of Si Sa Ket but rose again in 2017 and 2018 due to an increase in total cases from the 2017 outbreak in the province. Since 2019, most areas in the two provinces have seen a steady decrease in the proportion of all-case API density from baseline.

In 2013, there was a 0.34 maximum increase in the proportion of indigenous API density from baseline in villages around the Buntharik-Na Chaluai border (Fig. 13B). From 2017 to 2019, many areas in the south of Si Sa Ket, where the 2017 outbreak cases were largely confined, and in the southeast of Ubon experienced a large increase in the proportion of indigenous API density from baseline. There was a maximum 0.73 increase in Khun Han in 2017, and 1 and 0.46 increases in Kantharalak in 2018 and 2019, respectively.

**Fig. 14:**
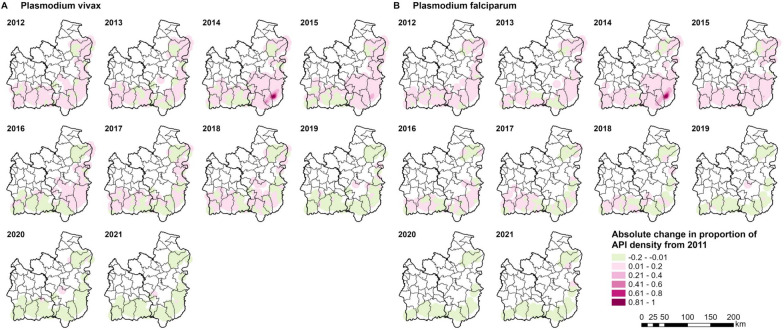
Absolute change in proportion of *Plasmodium* (**A**) *vivax* and (**B**) *falciparum* API density from 2011. For each parasite species, the proportion of API density was calculated by dividing each cell value by the maximum density value for that parasite species

The absolute changes in the proportions of *P. vivax* and *P. falciparum* API density were closely comparable (Fig. 14). The proportion of API density of cases from both parasite species increased from baseline in many areas between 2012 and 2015, particularly in Ubon. The increase was highest in 2014 (at 0.97 for *P. vivax* and 0.997 for *P. falciparum*) and 2015 (0.4 for *P. vivax* and 0.23 for *P. falciparum*) in Buntharik district. From 2016 to 2018, the proportion of API density of both reduced in many areas, although in some it remained above baseline. From 2019 onwards, the proportion of API density dropped below baseline in most areas.

## Discussion

### Indigenous transmission and case classification

From 2011 to 2021, there has been an overall reduction in the number of malaria cases reported in Si Sa Ket and Ubon Ratchathani provinces in Thailand, despite a large outbreak in Ubon during this period. An increasing proportion of those cases were classed as indigenous to the patient’s village of residence. While the proportion of all-cases API has reduced to below baseline, the proportion of indigenous API remained above baseline throughout the past decade. There are likely to be multiple factors contributing to this, including changes to surveillance strategies and dynamic human processes. The introduction of the 1-3-7 surveillance strategy and updated online reporting dashboard in the eMIS in 2016 [10] coincided with a gradual increase in the proportion of cases classified such that from 2017, at least 50% of reported cases were classified each month, increasing to more than 75% in 2021. The increase in the number and proportion of indigenous malaria cases in both provinces may therefore represent improved reporting rather than a true increase in malaria transmission within villages.

There were few village indigenous hot spots compared to subdistrict indigenous hot spots. During the outbreak in the southeast of Ubon, there were high-confidence subdistrict hot spots but no village hot spots. This pattern is likely due to low rates of case classification. No indigenous cases were reported in some months of 2013–2015 in Ubon, but there were high numbers of unclassified cases: 56.1% of all cases in 2014 were unclassified. This improved as case numbers dropped. A likely explanation for this transient reduction in reporting quality is that healthcare workers were overwhelmed with cases and therefore unable to complete the epidemiological surveillance forms. Individual village/clusters risk stratification data suggests that indigenous malaria transmission was ongoing in villages or clusters of houses in villages at the time. Prior to 2016, an A1 village/cluster was defined as a “perennial transmission village or hamlet where indigenous cases are reported at least 6 months out of the year”, while an A2 village/cluster was where “indigenous cases are reported fewer than 6 months out of the year” [21]. If a village/cluster had not reported indigenous transmission for at least 3 years, but primary vectors were found or conditions were felt to be favourable for breeding, it was classed as B1 [21]. In the risk stratification dataset, in Ubon, 14 villages/clusters were classed as A1 in 2013, 15 in 2014, and 6 in 2015. There were 29 A2 villages/clusters in 2013, 33 in 2014, and 126 in 2015. This increase in the number of high-risk villages/clusters implies that the presence of indigenous transmission was being acknowledged despite only two village indigenous cases having been reported in the province in 2014, one of which was within the study districts. Accounting for this, and the high proportion of unclassified cases, it was assumed that the lack of village hot spots compared to subdistrict hot spots was due to low rates of classification rather than a true absence of village indigenous malaria transmission. It was also assumed that the missing, unclassified data is balanced and representative of the other proportions.

### Drivers of malaria trend

Mobile and migrant populations (MMPs) pose major challenges to malaria prevention and control programme in the country [7]. The study area borders Cambodia and Lao and cross-border surveillance is complicated by high population mobility, which is associated with the importation of malaria parasites [22]. Data from Cambodia and Lao were not available to us for inclusion in this analysis, and there was no information on the origin of imported cases. However, in both these countries, malaria incidence immediately across the border from the study area has steadily decreased and was close to zero by 2021 (https://www.who.int/initiatives/mekong-malaria-elimination-programme/epidemiology-summaries). Human processes in areas covered by plantations and tropical forests may lead to malaria importation and subsequently local transmission [23]. Community members in Si Sa Ket and Ubon participate in different agricultural activities in rice fields [24], rubber plantations [24, 25], cassava plantations [25], and forest-going activities [24].

There was a large peak in unclassified cases between 2014 and 2016 in Ubon: the number of reported malaria cases rose from 1081 in 2013 to 8321 in 2014, an increase of 670%. This outbreak, confined largely to Buntharik, Na Chaluai, and Nam Yuen districts in Ubon, which share a forested border with Lao PDR to the east, was anecdotally related to an increase in the price of rosewood (*Dalbergia cochinchinensis Pierre ex Lannes*), which led to an increase in forest-going activities to harvest the wood [19, 26]. An entomological study of *Anopheles* mosquitoes in 8 village sentinel sites at the time found no *Plasmodium* species, although few primary vectors were collected [19]. This implies that infection was occurring outside of the villages and within the forest. The 2017 outbreak in the southern districts of Si Sa Ket bordering Cambodia was also suggested to have been driven by an increase in forest-related activities among rubber tappers and military personnel, although the cause of the outbreak was unknown [20].

In separate work conducted in the same area and timeframe as paper, the relationship between forest cover, API, and reproductive number under control (R_c_) was investigated. The findings indicated that the villages in densely forested subdistricts and those with higher forest cover within a 5 km radius were associated with higher indigenous API and R_c_ [27]. Controlling transmission around cases imported from the forest and other regions is important: the locations of stable hot spots for indigenous cases and all cases in the present study are very similar. This suggests that indigenous cases may be largely introduced cases fed by non-indigenous infections, and so curbing local transmission also relies on reducing importation, which was high during the 2014 outbreak in Ubon. Were a similar situation to arise in the future, this would present a challenge for surveillance efforts: forest-goers with fever may be hesitant to present to healthcare facilities or disclose their movements if they have been engaging in potentially illegal forest-going activity. Strategies targeting forest-goers such as portable ITNs and asymptomatic testing have variable uptake [24]. During the 2017 outbreak in Si Sa Ket, while overall ITN usage was high, it was sub-optimal among travellers who slept in forested areas [20]. Forest goers in Si Sa Ket and Ubon reported facing limitations in using preventive measures, such as repellents, coils, and mosquito nets, in forest settings when working or resting [24].

The majority of infections over the study period were *P. vivax*, although there were high *P. falciparum* case numbers during the 2014 outbreak in Ubon. Since 2016, there has been a marked reduction in the proportion of *P. falciparum* infections and hot spots*.* In contrast, while *P. vivax* hot spots have become less numerous over time, they persisted throughout the study period in parts of both provinces. The proportions of each species during the outbreak years were similar to reports across the border in Lao PDR in 2013–2016 in a study area including Moonlapamok and Sukhuma districts, which border Na Chaluai and Buntharik districts in Ubon [28]. It is important to be aware of the species mixes, as different *Plasmodium* species present specific elimination challenges. Anti-malarial drug resistance is a particular concern in the GMS where strains of artemisinin and ACT-resistant *P. falciparum* circulate. Chloroquine resistance is infrequently present in *P. vivax* [29]. Vivax presents an additional challenge to elimination efforts due to the potential for relapse from the dormant liver stages [30]. While radical cure with primaquine can help to prevent this recurrence, there is a risk of haemolysis in people with G6PD deficiency, which is estimated to affect 8–24% of the population in Northeast Thailand [31]. The recent introduction of point-of-care G6PD testing has increased access to primaquine, but a significant proportion of the population cannot safely take the full dose.

A recently published geospatial modelling study in Si Sa Ket and Ubon identified forest-based occupations and travel through high-risk areas for work as potential drivers of local malaria transmission [32]. The study also found that northern districts in Ubon near the forested Thai-Laotian border had a high probability of malaria occurrence. The present study identified *P. falciparum* subdistrict and village hot spots in these areas in 2021. Demographic, behavioural, and environmental factors are syndemic contributors to malaria transmission [23]. Environmental changes, such as upticks in temperature, humidity and precipitation, also contribute to elevated risk of malaria [33], particularly in regions where the disease has been eliminated [22].

### Implications for public health efforts

The use of malaria terminology can vary between programmes [3], but applying flexible definitions allows examination of data on different levels depending on intervention scale. The WHO defines indigenous malaria as a “case contracted locally with no evidence of importation and no direct link to transmission from an imported case” [3]. However, a specific definition of local is not given, and use of the term varies [34]. The time frame within which someone is considered at highest risk of being an imported case following travel to an endemic area also varies, from 10 days to 3 months [35]. The Thai DVBD defines an indigenous case as “a patient who contracted malaria in the village where the patient lived during infection period” [10] and states no specific time frame. A malaria infection acquired anywhere else is classed as imported. In this study, malaria cases were stratified based on likely location of infection in two ways: firstly, locally acquired was defined as acquired within the village of residence only, per the DVBD definition; and secondly, where locally acquired was defined as acquired within the village or the subdistrict of residence. These stratifications reflect both the level of interventions, which is tailored to individual villages/clusters [2], and the high level of mobility in at-risk forest-going populations [36], which often includes moving beyond the village. There were high-confidence indigenous hot spots in 2013 and 2014 in the northeast of Ubon which were not identified in the village-level analysis, indicating high levels of transmission within the subdistrict but outside of the home village.

The key hot spots of indigenous transmission identified in this analysis appeared to be stable, and these hot spots contributed high numbers of malaria cases. Stable hot spots have been found in previous studies to be highly predictive of future malaria risk [37, 38], which supports the spatial targeting of receptive villages/clusters with interventions. There were high-confidence hot spots of indigenous malaria transmission at the village and subdistrict levels in the south of Si Sa Ket and the southeast of Ubon for much of the study period. The statistical significance of these hot spots remained high despite declining case numbers, implying that they were very stable [38]. Targeting malaria foci in elimination and post-elimination settings has been recommended by the WHO [39]. However, there is mixed evidence regarding whether geographical malaria hot spots should always be targeted for interventions in near-elimination settings [40]. A geospatial modelling study in Zimbabwe found that hot spots identified from case data had a strong positive correlation with *Anopheles arabiensis* habitats [41]. A study in the Western Kenyan Highlands found that targeted interventions had a modest impact on parasite prevalence, and this did not affect prevalence in areas adjacent to the hot spots [42]. Stresman et al*.* [40] recommended that where the bulk of transmission occurs away from settlements, targeting of behavioural traits rather than geographic locations would be advantageous. This is consistent with the current approach taken by the DVBD, which specifies that villages/clusters which are not active foci (i.e. which do not have ongoing indigenous transmission) should implement educational strategies for night-time forest-goers [2], thus aiming to reduce the importation of cases. The findings of this study support the country’s current focus on targeting high-risk groups as well as the spatial targeting of villages/clusters that are active foci. They also highlight the need to prioritize malaria service provision in hot spots which are home to farmers, forest-going populations, and MMPs at risk of contracting malaria. The continued use of malaria prevention measures that are tailored to risks and preferences of forest-going populations is important in low-transmission settings [24]. Moreover, as the global climate evolves, adaptive strategies that address climatic impacts on malaria risk and control programmes should be developed [43].

### Strengths and limitations

Here high-quality malaria surveillance data over a dynamic 10-year period were analyzed, including malaria outbreaks and changes to the national malaria strategy. Manually geolocated village coordinates were used for the spatial analyses. The factors contributing to the decline in overall cases and increase in indigenous cases were considered, and how these are relevant to future control efforts.

Important limitations include a lack of data on the prevalence of asymptomatic infection from active surveillance, which is currently offered to close contacts of malaria cases, and before and during the rainy season in active foci [2]. Although the cases with incomplete village location data constituted a relatively small proportion of the raw malaria case data, excluding them might have led to underrepresentation of total malaria cases. Calculated API values might have been slightly underestimated, and there could have been some distortion in the spatial distribution of API, possibly leading to misidentifying true hot spots or falsely identifying them. Many cases were not classified by likely location of infection between 2012 and 2016, but from 2017 onwards there was a much greater proportion of cases classified. Comparisons between earlier and later data should, therefore, be made with caution due to differing levels of completeness. There was also the potential for inaccurate geolocation of cases, for example in the case of recrudescence/relapse of previous infection, or inaccurate reporting of travel history to those performing case investigations. It was also not possible to differentiate potential recurrences of *P. vivax* from new infections. Securing comprehensive, accurate surveillance data will be crucial for informing effective public health actions and sustaining malaria elimination efforts in the region.

Village GPS coordinates used in this study were collated from different sources, including manual collection by the study team in the field, as described in Additional file 1. Due to the quantity of villages with no location data and the amount of time required to locate them, it was decided to include only the villages in the districts that comprised 95% of the cases in each province in village-level analyses. Compiling a list of village GPS coordinates of quality that is interoperable with other existing datasets, such as malaria surveillance data and population counts, would be of great usefulness for future disease mapping efforts. Ideally, such a list should be centrally managed by the government as a single high-quality master list for all purposes, including those beyond health.

Future work should utilize information sharing with programs in Lao PDR and Cambodia to allow better understanding of cross-border epidemiology and work towards the shared goal of elimination across the GMS. Now that case numbers are low enough to permit it, spatiotemporal modelling techniques could be used to form likely links between cases and determine whether locally transmitted cases are largely indigenous or introduced by importation [8, 44]. Optimizing climate and environmental data, along with strengthening collaboration with non-malaria and non-health sectors such as the Ministry of Environment and other relevant entities, is important for evaluating malaria risk and transmission [22].

## Conclusion

In Northeast Thailand, there has been a large, non-linear decline in the number of malaria cases reported between 2011 and 2021 and over time an increasing proportion of those cases have been classed as indigenous to the patient’s village of residence. It is likely that the increase in the proportion of indigenous cases was the product of the introduction of case investigation, as there has been a reducing proportion of unclassified cases reported. There were stable hot spots of ongoing transmission in the forested border areas where intervention is complicated by remote location and high-risk forest-going activities. Hot spots of indigenous and all cases were in similar locations, indicating that the non-indigenous cases may have been feeding the former. It was not possible to assess this due to the large number of cases. The majority of identified species of infection were *P. vivax,* but there was a spike in *P. falciparum* cases during a large outbreak in Ubon Ratchathani in 2014. There is a risk that there could be similar future outbreaks if demographic and behavioural changes increase contact with transmission reservoirs in the forest, or if environmental changes favour malaria transmission. The findings of this study emphasize the importance of targeting high-risk populations and prioritizing active foci. Stable hot spots near forested international borders should be closely monitored to mitigate the risk of transmission. Despite success in reducing case numbers over the past 10 years, ongoing concentrated surveillance, tailored and adaptive interventions, along with strengthened cross-border collaborations, are required in order to reach malaria elimination goals in this region.

### Supplementary Information

Additional file 1: Methods. Details of sources used for village GPS coordinates, the analysis of village hot spot stability, and the analysis of absolute change in the proportion of API density from baseline.

Additional file 2: Number of malaria cases by region by parasite species from 2011 to 2021. Table presenting the number of malaria cases by region by parasite species from 2011 to 2021.

## Data Availability

The population counts per village datasets supporting the conclusion of this article are available in the Official statistics registration systems repository, https://stat.bora.dopa.go.th/new_stat/webPage/statByYear.php [13]. The village GPS coordinates collected by our field staff and in Street View in Google Maps are available from the corresponding author on reasonable request. The Thailand administrative boundaries dataset used in the current study is publicly available at https://data.humdata.org/dataset/cod-ab-tha [14]. The administrative boundaries datasets for other countries are publicly available at https://gadm.org/download_country.html [15]. The malaria surveillance datasets and village GPS coordinates in the DVBD surveillance database analysed during the current study are not publicly available as they belong to the Department of Disease Control, Ministry of Public Health, Thailand, but are available on reasonable request.

## Abbreviations

WHO: World Health Organization
GMS: Greater Mekong Subregion
Lao PDR: Lao People’s Democratic Republic
DVBD: Division of Vector-Borne Diseases
ACT: Artemisinin-based combination therapy
eMIS: Electronic Malaria Information System
SPR: Slide positivity rate
API: Annual Parasite Index
GPS: Global Positioning System
RDT: Rapid diagnostic test
Ubon: Ubon Ratchathani
MMPs: Mobile and migrant populations
R_c_: Reproductive number under control
ITNs: Insecticide-treated nets
G6PD: Glucose-6-phosphate dehydrogenase
