# Supplementary material for: Mapping malaria transmission foci in Northeast Thailand from 2011 to 2021: approaching elimination in a hypoendemic area
Source: Malar J. 2024 Jul 17;23:212. doi: 10.1186/s12936-024-05026-6 (PMC11253324; doi:10.1186/s12936-024-05026-6)
Supplement: Supplementary file 1 — Additional file 1: Methods. Details of sources used for village GPS coordinates, the analysis of village hot spot stability, and the analysis of absolute change in the proportion of API density from baseline. [file 12936_2024_5026_MOESM1_ESM.docx]

**Methods**

**Village list**

Of 5,447 villages listed in the two provinces, there were 5,343 villages and 104 central house registrations. A central house registration is an entry with a village code ending with 00 which is used to list individuals whose names are not registered to a house(1). One duplicate village was found and removed. For the subdistrict-level analysis, the population counts of the remaining 5,446 entries (2,638 villages and 41 central house registrations in Si Sa Ket; 2,704 villages and 63 central house registrations in Ubon Ratchathani) were aggregated by subdistrict for API calculation. For the village-level analysis, only the villages were used.

Four cases (0.05%) in Si Sa Ket and 77 cases (0.47%) in Ubon Ratchathani had a residence village code ending with 00, indicating that the Moo (village) number was missing; 113 cases (1.42%) in Si Sa Ket and 418 cases (2.57%) in Ubon Ratchathani had no recorded residence village code; and 43 (0.27%) cases in Ubon had residence village codes that did not match with a village code in the list of villages.

**Village GPS coordinates**

Three sources were used for village GPS coordinates: (1) manually collected GPS coordinates by our field staff covering all villages in Phu Sing district in Si Sa Ket, and Nam Yuen, Na Chaluai, and Buntharik districts in Ubon; (2) manually validated GPS coordinates in the DVBD surveillance database covering 26 of 771 (3.4%) of villages in Kantharalak district in Si Sa Ket, and Det Udom, Sirindhorn, Khong Chiam, and Si Mueang Mai in Ubon Ratchathani; and (3) Street Vew in Google Maps for the 583 remaining villages.

**Getis ord statistic**

This tests for local spatial autocorrelation; it identifies statistically significant local clusters of high values (hot spots) and low values (cold spots) in the spatial distribution of a variable (the API in this analysis). It compares whether the observed local sum of spatially weighted values for the feature and its neighbour (the sum of API values for a subdistrict or for a village and its neighbour, weighted by how close they are) is significantly different from the global average (the average API for all subdistricts or for all villages) under the null hypothesis of complete spatial randomness; i.e., what would be expected if values were randomly distributed across the study area. The statistic yields a z-score (Gi*) and p-value for each feature. The z-score is the standard deviation, with a large positive z-score indicating spatial clustering of high values and large negative z-score indicating spatial clustering of low values. The p-value is the probability that the observed spatial clustering is the result of random chance. Using 90%, 95%, and 99% confidence levels, hot spots are identified if they have a p-value smaller than 0.10, 0.50, and 0.01 and a z-score greater than 1.65, 1.96, and 2.58 (smaller than −1.65, –1.96, and –2.58 for cold spots), respectively.

**Village hot spot stability**

The density of the number of years was estimated using the Kernel Density tool in ArcGIS Pro version 3.1.0. The tool calculates a magnitude-per-unit area by fitting a smoothly curved surface over each observation using the quartic kernel function. The surface value is maximal at the location of the observation (village) and decreases as it approaches the given bandwidth. The density at each output raster cell is estimated by summing up the values of all the kernel surfaces that overlay the raster cell centre. The tool parameters were set as the bandwidth at 8,547 metres; the area unit of the output density in square kilometres; and the output cell size at 500 metres. The resultant density values were then converted to proportions by dividing each cell value by the maximum density value for the corresponding case type.

**Absolute change in the proportion of API density from baseline**

For each case classification and parasite species, the density of API was calculated at and from each village each year using the Kernel Density tool in ArcGIS Pro version 3.1.0 with the same parameter values as the village hot spot stability analysis. The proportion of API density was calculated by dividing each cell value by the maximum density value from all years for the corresponding case type. To calculate the absolute change from baseline for each case type each year, the baseline proportion for that case type was subtracted from the proportion of API density for that case type that year.

**References**

1. House and housing registration [Internet]. [cited 2023 Sep 20]. Available from: https://www.bora.dopa.go.th/CallCenter1548/index.php/menu-population/13-service-handbook/population/23-population-house
