# Supplementary material for: Mapping malaria transmission foci in Northeast Thailand from 2011 to 2021: approaching elimination in a hypoendemic area
Source: Malar J. 2024 Jul 17;23:212. doi: 10.1186/s12936-024-05026-6 (PMC11253324; doi:10.1186/s12936-024-05026-6)
Supplement: Supplementary file 2 — Additional file 2: Number of malaria cases by region by parasite species from 2011 to 2021. Table presenting the number of malaria cases by region by parasite species from 2011 to 2021. [file 12936_2024_5026_MOESM2_ESM.docx]

Number of malaria cases by region by parasite species from 2011 to 2021

| Year | Si Sa Ket + Ubon | | | | Si Sa Ket | | | | Ubon | | | |
| --- | --- | --- | --- | --- | --- | --- | --- | --- | --- | --- | --- | --- |
|  | All | *P. v.* | *P. f.* | Other | All | *P. v.* | *P. f.* | Other | All | *P. v.* | *P. f.* | Other |
| 2011 | 1061 | 881 | 180 | 0 | 652 | 560 | 92 | 0 | 409 | 321 | 88 | 0 |
| 2012 | 2491 | 1563 | 547 | 381 | 1479 | 1017 | 268 | 194 | 1012 | 546 | 279 | 187 |
| 2013 | 2261 | 1338 | 541 | 382 | 1180 | 835 | 209 | 136 | 1081 | 503 | 332 | 246 |
| 2014 | 9219 | 4613 | 3110 | 1496 | 898 | 520 | 283 | 95 | 8321 | 4093 | 2827 | 1401 |
| 2015 | 4466 | 2627 | 1598 | 241 | 1197 | 386 | 659 | 152 | 3269 | 2241 | 939 | 89 |
| 2016 | 1112 | 706 | 293 | 113 | 353 | 161 | 143 | 49 | 759 | 545 | 150 | 64 |
| 2017 | 1208 | 883 | 238 | 87 | 937 | 716 | 201 | 20 | 271 | 167 | 37 | 67 |
| 2018 | 1362 | 1040 | 212 | 110 | 888 | 712 | 169 | 7 | 474 | 328 | 43 | 103 |
| 2019 | 306 | 247 | 39 | 20 | 185 | 152 | 31 | 2 | 121 | 95 | 8 | 18 |
| 2020 | 48 | 44 | 2 | 2 | 30 | 28 | 2 | 0 | 18 | 16 | 0 | 2 |
| 2021 | 36 | 33 | 2 | 1 | 26 | 25 | 0 | 1 | 10 | 8 | 2 | 0 |
| All | 23570 | 13975 | 6762 | 2833 | 7825 | 5112 | 2057 | 656 | 15745 | 8863 | 4705 | 2177 |

All = *P. vivax* (*P. v.*) + *P. falciparum* (*P. f.*) + *P. malariae* + *P. ovale* + mix + unknown
